# Chiropractic management of dominating one-sided pelvic girdle pain in pregnant women; a randomized controlled trial

**DOI:** 10.1186/s12884-017-1528-9

**Published:** 2017-09-29

**Authors:** Anne Marie Gausel, Inger Kjærmann, Stefan Malmqvist, Knut Andersen, Ingvild Dalen, Jan Petter Larsen, Inger Økland

**Affiliations:** 1 The Norwegian Centre for Movement Disorders, Stavanger University Hospital, Stavanger, Norway; 2 Sundbybergskliniken, Sundbyberg, Sweden; 3 Department of Research, Section of Biostatistics, Stavanger University Hospital, Stavanger, Norway; 4 Department of Obstetrics and Gynecology, Stavanger University Hospital, Stavanger, Norway

**Keywords:** Pregnancy, Manual therapy, Sick leave, Subgroups, SMS track

## Abstract

**Background:**

The aim of this study was to investigate the outcome of chiropractic management for a subgroup of pregnant women with dominating one-sided pelvic girdle pain (PGP).

**Methods:**

The study population was recruited from a prospective longitudinal cohort study of pregnant women. Women reporting pelvic pain (PP), and who were diagnosed with dominating one-sided PGP after a clinical examination, were invited to participate in the intervention study. Recruitment took place either at 18 weeks, or after an SMS-tracking up to week 29. The women were randomized into a treatment group or a control group. The treatment group received chiropractic treatment individualized to each woman with regards to treatment modality and number of treatments. The control group was asked to return to conventional primary health care. The primary outcome measure was new occurrence of full time and/or graded sick leave due to PP and/or low back pain. Secondary outcome measures were self-reported PP, physical disability and general health status. Proportion of women reporting new occurrence of sick leave were compared using Chi squared tests. Differences in secondary outcome measures were estimated using linear regression analyses.

**Results:**

Fifty-Six women were recruited, and 28 of them were randomized into the treatment group, and 28 into the control group. There was no statistically significant difference in sick leave, PP, disability or general health status between the two groups during pregnancy or after delivery.

**Conclusion:**

The study did not demonstrate superiority of chiropractic management over conventional care for dominating one-sided PGP during pregnancy. However, the analyses revealed wide confidence intervals containing both positive and negative clinically relevant effects**.**

**Trial registration:**

The study was registered in ClinicalTrials.gov (NCT01098136; 22/03/2010).

## Background

Pelvic pain (PP) is a common complaint during pregnancy, and about 50% of pregnant women are troubled with pain in the pelvic region during pregnancy [1–3]. The pain varies in intensity and duration, and the women experience different degrees of disability [4, 5]. These complaints are a frequent cause of sick leave during pregnancy [6, 7]. Also, we found in a previous study that 16% of women with PP during pregnancy reported persistent pain that affected their daily life activities 3–6 months after delivery [8].

A large number of different terms have been used to describe PP during pregnancy, such as lumbopelvic pain, sacroiliac pain and pelvic instability [4, 5], but there are little consensus on definition and classification. Therefore, it is difficult to compare therapies, and to assess their effect on PP in pregnancy.

In Norway, most clinics in the primary health care system offer treatment for women with PP during pregnancy. Manual therapy is a common treatment modality, yet its evidence is limited, and the studies showing that chiropractic care during pregnancy is safe and might relieve symptoms are of low and medium quality [4, 9–11]. Moreover, a recent Cochrane review, investigating interventions for preventing and treating PP and back pain in pregnancy, found no studies of high quality to prove that spinal manipulation has a positive effect on PP [12]. The European guidelines for the diagnosis and treatment of pelvic girdle pain (PGP) also conclude that there is a need for more studies on the effect of manipulative treatment of PP during pregnancy [4].

To our knowledge, none has so far investigated the effect of chiropractic treatment on specific subgroups of PP. This is relevant because the diagnostic picture of PP is complex. By isolating subgroups of pregnancy-related PP it might be possible to differentiate the women who could favor from chiropractic treatment from those who will not.

The aim of this study was to investigate the effect of chiropractic management for a subgroup of pregnant women with dominating one-sided PGP in a randomized controlled trial (RCT).

## Methods

### Study design

This is a randomized controlled intervention study of pregnant women, conducted in an obstetric and chiropractic outpatient clinic at Stavanger University Hospital, Norway.

The participants were recruited from a prospective longitudinal cohort study, which investigated the incidence and the course of PGP during pregnancy, using questionnaires, clinical examination and SMS-tracking. All women admitted for the routine second-trimester ultrasound examination at Stavanger University Hospital were asked to participate in the cohort study.

Inclusion criteria for participation in the prospective cohort study were a low risk, singleton pregnancy and comprehension of the Norwegian language. At the routine ultrasound examination at 18 weeks of pregnancy, all women willing to participate in the prospective cohort study were asked to sign an informed consent, to fill in questionnaires containing demographic and clinical information. Furthermore, women reporting PP verified by pain drawings were invited to meet for a clinical examination performed by a chiropractor.

As part of the prospective cohort study, all women were followed by means of an SMS track survey [13–15]. This consisted of a question that every Sunday was sent to the participant’s mobile phone, asking about the number of days with bothersome PP experienced during the last week. Those without PP at baseline were asked to meet for clinical examination if they, according to the SMS track survey, reported more than four days with PP and were still less than 29 weeks pregnant. Only women diagnosed with dominating one-sided PGP after the clinical examination were invited to participate in this RCT.

For all symptomatic women in the cohort, the examination procedure at baseline, including the questionnaire package, was repeated at 30 weeks of pregnancy and six weeks after delivery. The information collected around week 18 will be referred to as baseline data.

In this sub-study, we included the women that were diagnosed with dominating one-sided PGP after a clinical examination. The women were randomized into a treatment group or a control group.

The data were collected in the period March 2010 − December 2010, and the women were followed from inclusion around pregnancy week 18 until six weeks after delivery. The study was approved by the Regional Ethics Committee of Western Norway (no. 2010/174), adheres to the CONSORT guidelines regarding RCTs and is registered in ClinicalTrials.gov (NCT01098136; 22/03/2010).

### Study population

**Fig. 1:**
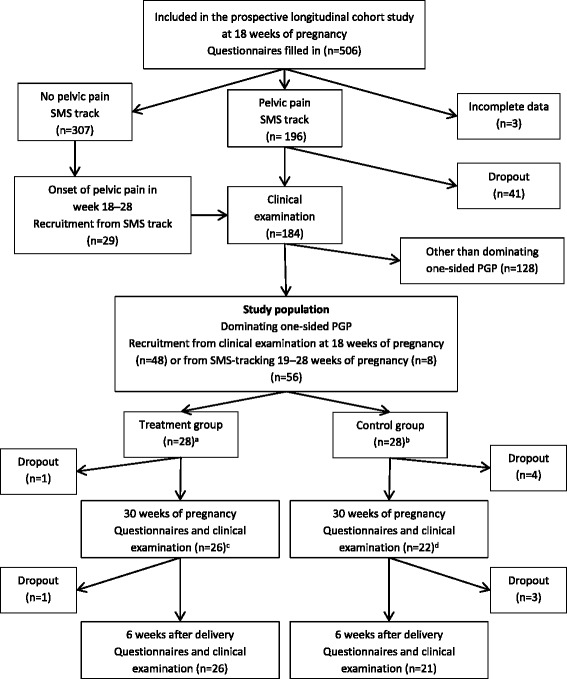
Flow chart of the inclusion process into the randomized controlled trial. **a** 3 women did not meet for scheduled appointment for treatment and did not respond to several attempts of contact. They were included in the intention-to- treat analyses, but excluded from per-protocol subanalyses. **b** 7 women underwent chiropractic treatment as conventional care. They were included in the control group in the intention-to-treat analyses but excluded in the per protocol subanalyses. **c** 1 missing observation. The woman did not fill in questionnaires nor meet for clinical examination at 30 weeks of pregnancy, but returned to the study six weeks after delivery. **d** 2 missing observations. The women woman did not fill in questionnaires nor meet for clinical examination at 30 weeks of pregnancy, but returned to the study six weeks after delivery

In total, 506 women were recruited for the prospective cohort study. Out of these, 196 (39%) participants reported pain in the pelvic region at inclusion. After the clinical examination, 48 women were diagnosed with dominating one-sided PGP, and included in the intervention study. Additionally, eight women recruited from the SMS-tracking before 29 weeks´ pregnancy were diagnosed with dominating one-sided PGP and included in the study, i.e. in total 56 women were randomized into the treatment group (n = 28) or the control group (n = 28). Figure 1 shows the inclusion process into the RCT.

### Questionnaires and clinical examination

At baseline, all the women answered questions regarding demographic information, sick leave, previous illnesses and treatments, current symptoms, pain location and duration, workload, possible co-morbidities, and filled in the Norwegian version of Oswestry Disability Index questionnaire (ODI), and the EQ-5D health questionnaire (EQ-5D) [8]. Intensity of PP was examined using a numeric rating scale (NRS). The women were asked to retrospectively report average PP. The characteristics of the different questionnaires are described in detail elsewhere [8].

The physical examination included a functional analysis of the lumbar spine and pelvis, and a neurological examination of the lower extremities. In addition, several specific orthopedic tests were performed. These tests are considered to have a high specificity for PGP and are recommended in the European guidelines [4]. We included posterior pelvic pain provocation test, Patrick’s Faber test, palpation of the long dorsal sacroiliac ligament and Gaenslen’s test. In addition, symphysis pain was assessed using palpation of the symphysis and modified Trendelenburg test of the pelvic girdle. Active straight leg raise was also performed as a functional pelvic test. A PGP diagnosis was achieved if the women reported pain in the vicinity of the pelvic joints, and had reproducible pain after one of the specific pain provocation tests listed above, and if a lumbar cause of pain were excluded. Women with a one-sided positive posterior pelvic pain provocation test, a bilateral negative Lasègue test, and a pain drawing indicating one-sided pelvic symptoms were considered to have dominating one-sided PGP.

### Intervention

The women were randomized into a treatment group or a control group, using a closed envelope. The envelope contained a complete ID-code, and was handed out after the first clinical examination. Women with an ID-code that ended with an even number were asked to join the intervention group, whereas women with and ID-code that ended with an uneven number were asked to return to conventional health care. The examiner was blinded for which group the women belonged to at the clinical examinations. Additional blinding or sham treatment (placebo) was not implemented.

For the treatment group, the intervention consisted of manipulation, mobilization, soft tissue treatment, exercises, and advices chosen by the chiropractor to fit each participant individually. The frequency and number of visits were also determined by the chiropractor. The chiropractic treatment was conducted in two different private clinics, by five different chiropractors. The chiropractors were randomly chosen, and willing to contribute to the study. They were experienced generalists, not specialized in treatment of pregnant women and were given information about the study in order to keep to the protocol.

The women in the control group were asked to return to conventional primary health care without any restrictions or recommendations.

### Outcome measures

The primary outcome measure was new occurrence of full time and/or graded sick leave due to PP and/or low back pain (LBP), in the periods 19 − 30 weeks and 31 − 36 weeks of pregnancy, among the women who did not report sick leave for any reason in week 1 − 18. In Norway, working women are offered maternity leave paid by the Norwegian Labour and Welfare Service (NAV), starting at 37 completed pregnancy weeks.

Secondary outcome measures were self-reported pain intensity, as an average of the periodical NRS scores, physical disability as measured by the ODI questionnaire and general health as measured by the EQ-5D questionnaire.

### Statistical analysis

Three of the 28 women (11%) that were randomized into the treatment group did not meet for treatment. In the control group, seven women (25%) reported having chiropractic treatment as part of conventional care. Because of this, we conducted two types of analyses, an intention-to-treat analysis and a per-protocol analysis. Overall, results from the per-protocol analysis did not differ substantially from those from the intention-to-treat analysis, and therefore only the intention-to-treat results are presented.

All statistical analyses were performed in SPSS (PASW Statistics 21). Descriptive statistics are given as means and standard deviations (SDs), and as counts and percentages. Proportion of women reporting new occurrence of sick leave in the treatment and the control group were compared using Chi squared tests. Relative risks with 95% CIs were estimated using the online statistical calculator at http://vassarstats.net/odds2x2.html. For the secondary outcomes, treatment effects were estimated using linear regression analysis, including the respective baseline measurements as covariates. In the next instance, possible confounders that were not satisfactorily balanced at baseline, i.e., exercise before and during pregnancy and PP one year before pregnancy (Table 1), were included in the models.

**Table 1:** Demographic and clinical features for the treatment and control group at baseline. Given as counts (%) unless otherwise stated

	Treatment group *n* = 28	Control group *n* = 28
Age at inclusion (years), mean (SD)	28.9 (4.5)	29.9 (4.8)
Age ≥ 30	13 of 28 (46)	14 of 28 (50)
Primiparous	16 of 26 (62)	15 of 27 (56)
Education length (years)^a^, mean (SD)	14.7 (4.0)	14.8 (3.1)
More than 12 y education baseline	21 of 27 (78)	21 of 25 (84)
Heavy workload baseline	6 of 28 (21)	6 of 28 (21)
BMI before pregnancy, mean (SD)	23.4 (3.1)	24.2 (4.0)
Depressed in pregnancy	1 of 27 (4)	1 of 28 (4)
Exercise before pregnancy	5 of 26 (19)	12 of 27 (44)
Exercise in early pregnancy (week 1 to18)	2 of 27 (7)	5 of 27 (19)
PP one year before pregnancy	9 of 27 (33)	4 of 27 (15)
PP and LBP in early pregnancy (week 1 to 18)	22 of 26 (85)	22 of 27 (82)
Sick leave in early pregnancy^b^ (week 1 to 18)	6 of 28 (21)	3 of 28 (11)

SD standard deviation BMI body mass index PP pelvic pain LBP low back pain

^a^n for education length is 27 and 25 for treatment and control group, respectively

^b^Only sick leave due to PGP and/or LBP

## Results

Out of the 28 women in the treatment group, 25 received chiropractic treatment. On average, they started treatment at week 23.1 (SD 2.1) and completed treatment at week 36.6 (SD 5.0). In total, they received between three and 15 treatments, with a mean of 10.3 (SD 3.6). The women received high-velocity, low-amplitude manipulative therapy to the lumbar spine and the sacroiliac joints, except for one, who underwent mobilization therapy that included low-velocity, passive movement within or at the limit of joint range. All participants had soft-tissue therapy, and 17 women also received information and a program on how to perform exercises at home.

Demographic information and clinical features for the treatment group and the control group are presented in Table 1. There were some baseline imbalances: the treatment group exercised less before and during pregnancy, and reported more PP one year before pregnancy, compared with the control group.

Table 2 shows the primary outcome measure, reported as new occurrence of sick leave in the periods 19 − 30 and 31 − 36 weeks. There was no statistically significant difference between the two groups. The treatment group reported 33% and 38% new occurrence of sick leave in the two periods, compared with 38% and 53% in the control group. The relative risk for new sick leave was 0.88 (95% CI, 0.39− 1.98) at 19 − 30 weeks, and 0.72 (95% CI, 0.36 − 1.45) at 31 − 36 weeks.

**Table 2:** New occurrence of sick leave due to PGP and/or LBP disregarded sick leave at baseline, and estimated effect of treatment

	Treatment group	Control group	RR	95%CI	*p*
Week 19 − 30, n (%)	7/21 (33)	8/21 (38)	0.88	0.39 − 1.98	0.75
Week 31 − 36, n (%)	8/21 (38)	10/19 (53)	0.72	0.36 − 1.45	0.36

*RR* relative risk, *CI* confidence interval

Secondary outcome measures and estimated effect of treatment are presented in Table 3. Both groups reported increased pain intensity at the follow-up visit during pregnancy, compared with PP at baseline. Adjusting for baseline pain, the treatment group reported somewhat lower PP in week 21 − 30 and week 33 − 40, compared with the control group. Oppositely, 0 − 6 weeks after delivery, the treatment group reported more pain than the control group. However, none of these differences were statistically significant.

**Table 3:** Estimated means of secondary outcome measures and estimated effect of treatment

	Treatment group Mean	95% CI	Control group Mean	95%CI	Mean difference^a^ β	95% CI	*p*
Pain intensity^b^, week 1 − 18	17.4 ^*n* = 26^	10.1 − 24.7	20.0 ^n = 28^	13.6 − 26.4			
Pain intensity^b^, week 21 − 30	42.7 ^*n* = 25^	33.5 − 51.8	46.4 ^*n* = 21^	37.3 − 55.6	−3.3	−15.1 − 8.5	0.58
Pain intensity^b^, week 33 − 40	40.3 ^*n* = 24^	27.9 − 52.8	44.2 ^n = 21^	29.8 − 58.5	−1.6	−19.4 − 16.3	0.86
Pain intensity^b^, week 1 − 6 after delivery	19.1 ^n = 24^	10.0 − 28.2	12.8 ^n = 21^	3.8 − 21.8	7.8	−4.9 − 20.4	0.22
ODI^c^, week 18	22.8 ^n = 26^	17.6 − 28.1	21.5 ^*n* = 26^	17.0 − 26.0			
ODI^c^, week 30	29.7 ^n = 25^	22.1 − 37.2	27.1 ^n = 21^	21.0 − 33.2	−0.9	−8.3 − 6.4	0.80
ODI^c^, 6 weeks after delivery	9.7 ^n = 25^	4.3 − 15.1	7.1 ^*n* = 20^	3.2 − 10.9	0.3	−4.9 − 5.4	0.92
EQ-5D^d^, week 18	64.9 ^n = 28^	59.2 − 70.7	62.0 ^n = 26^	55.3 − 68.6			
EQ-5D^d^, week 30	58.3 ^n = 26^	48.9 − 67.7	62.0 ^n = 21^	54.6 − 69.5	−3.3	−14.5 − 7.9	0.56
EQ-5D^d^, 6 weeks after delivery	84.7 ^n = 25^	77.8 − 91.6	86.8 ^n = 20^	78.6 − 95.1	−0.8	−11.1 − 9.4	0.87

^a^Results from linear regression, adjusting for the relevant outcome measured at baseline

^b^Pain intensity (numerical rating scale) with possible values 0 to 100, where 0 represents no pain and 100 represents most pain imaginable

^c^Oswestry disability index with possible values 0 to 100, where 0 represents no disability and 100 represents maximum disability possible

^d^Eurocol-5D with possible values −7 to 100, where −7 represents poorest health and 100 represents full health

*CI* confidence interval, *ODI* Oswestry disability index, *EQ-5D* Eurocol-5D

The reported disability was comparable for the two groups. Both groups reported a high degree of disability at 30 weeks and only minor disability at six weeks after delivery. The treatment group reported a worsened health status at 30 weeks, whereas the control group did not. Six weeks after delivery both groups reported an improved general health status.

Linear regression analysis with adjustment for the respective baseline measures showed no statistically significant difference between the two groups in any of the outcome measures, as shown in Table 3. Also, adjusting for the baseline imbalances in PP one year before pregnancy, and exercise before pregnancy and in early pregnancy (1 − 18 weeks), did not affect the conclusions. See Table 4.

**Table 4:** Estimated effect of treatment adjusted for baseline imbalances^a^

	Mean Difference^b^ β	95%CI	*p*
Pain intensity^c^, week 21 − 30	−0.4	−13.1 − 12.4	0.95
Pain intensity^c^, week 33 − 40	−2.7	−23.0 − 17.6	0.79
Pain intensity^c^, week 1 − 6 after delivery	5.4	−8.5 − 19.2	0.44
ODI^d^, week 30	−1.2	−9.2 − 6.8	0.76
ODI^d^, 6 weeks after delivery	−0.1	−5.3 − 5.2	0.97
EQ-5D^e^, week 30	−2.7	−16.3 − 10.9	0.69
EQ-5D^e^, 6 weeks after delivery	−1.3	−12.8 − 10.1	0.81

CI confidence interval ODI Oswestry disability index EQ-5D Eurocol-5D

^a^PP one year before pregnancy and exercise before and in early pregnancy

^b^Results from linear regression, adjusting for the relevant outcome measured at baseline

^c^Pain intensity (numerical rating scale) with possible values 0 to 100, where 0 represents no pain and 100 represents unbearable

^d^Oswestry disability index with possible values 0 to 100, where 0 represents no disability and 100 represents maximum disability possible

^e^Eurocol-5D with possible values −7 to 100, where −7 represents poorest health and 100 represents full health

Another observation from these regression analyses was that pain score reported at baseline was a predictor for pain in later pregnancy (week 21 − 30: R^2^ = 0.14, *p* = 0.009; week 33 − 40: R^2^ = 0.12, *p* = 0.020), but not for pain reported six weeks after delivery (R^2^ = 0.03, *p* = 0.30). The strongest associations were seen for disability, for which the baseline ODI score explained half of the variance in ODI, both at pregnancy week 30 (R^2^ = 0.50, *p* < 0.001), and at six weeks after delivery (R^2^ = 0.50, *p* < 0.001). Also for general health, the baseline measure was associated the same measure at 30 weeks of pregnancy (R^2^ = 0.24, *p* = 0.001), and at six weeks after delivery (R^2^ = 0.12, *p* = 0.022).

At the next follow-up consultation, the women were asked to recall any negative reactions, however, no serious or long-lasting adverse events was registered.

## Discussion

The aim of this study was to investigate the effect of chiropractic treatment for a subgroup of pregnant women with dominating one-sided PGP. We found no statistically significant difference in sick leave, pain, disability or general health status between the treatment group and the control group during pregnancy or after delivery.

There is limited research on the natural course of PGP during pregnancy. Typically, PGP begins by the end of the first trimester and reaches peak intensity between pregnancy week 24 and 36 [1, 2, 5, 16]. After delivery, the PGP resolves within three months in most cases [2, 4, 8, 16, 17]. This is in line with our findings, as both the treatment and the control group had worsening of symptoms from week 18 and onwards, and they reported less pain and disability and a better general health status six weeks after delivery.

Previous studies [4, 9, 11], including the latest Cochrane review on interventions for preventing and treating low-back and pelvic pain during pregnancy [12], have shown limited evidence for the effect of manipulative therapy for PP during pregnancy. There is some evidence that spinal manipulation improves pain and functioning in patients with chronic LBP [18], however, these results cannot be immediately transferred to apply for pregnant women, due to inherent biomechanical, physiological and hormonal changes.

Chiropractic treatment aims at manipulation and joint mobilization; however, the uncertain etiology is reflected in the variety of offered treatments. Adverse events following spinal manipulation during pregnancy are found to be relatively rare [10]. Nevertheless, treatment should not be performed over a longer period of time unless there is a positive response. This is in compliance with the recommendation that manipulation and joint mobilization may be used for symptomatic relief, but should only be applied for a few treatments [4].

This study represents a new approach to investigate the effect of chiropractic treatment, by including only a specific subgroup of PGP. Albert et al. have proposed that PGP could be divided into five subgroups, and they found that women with pain in all three pelvic joints had the worst prognosis regarding development of long term pain, whereas women with isolated symphysiolysis recovered shortly after delivery [17]. To our knowledge, no previous intervention study has been carried out on pregnant women with dominating one-sided PGP.

Pain in the pelvic region is affecting around 50% of all pregnant women, resulting in various degrees of disability and frequent sick leave [2, 4–7]. In a qualitative study from Sweden, it is emphasized that improved treatment of PGP is of importance to increase the quality of life of pregnant women [19]. In our study, 25% of the women in the control group underwent chiropractic treatment as part of conventional care, indicating a wish for some kind of therapy. It is possible that the women in the control group had been biased by the information about the study and therefore wanted to try chiropractic treatment for their PP.

There are several limitations in this study. Unfortunately, we managed to include a relative low number of women into the clinical trial, despite a substantial number of women were recruited to participate in the prospective cohort study. As a result, the confidence intervals are wide, containing both positive and negative clinically relevant effects. With a larger cohort we would probably get a clearer result. There were a relative high number of dropouts in the control group and seven women in the control group underwent chiropractic treatment as part of conventional care. Also, three women randomized to the treatment group did not meet for treatment. Additional analyses to correct for non-compliance did not substantially change the results.

Blinding or sham treatment was not performed. So far, an established method for blinding in studies where spinal manipulation is used does not, to our knowledge, exist [20]. A placebo or a specific alternative treatment for the control group might have prevented women in the control group from dropping out or seeking chiropractic care.

The chiropractors were told to perform necessary treatment to fit each patient individually. This can be considered to be a limitation to our study, and is diverging from the Guideline for Reporting Interventions on Spinal Manipulative Therapy [21]. However, the design of our intervention is equivalent to the treatment a woman would receive, consulting a random chiropractor for PGP during pregnancy.

Registration of adverse events following treatments were of poor quality in our study. The women were asked if they had experienced any side-effects or negative reactions at the next consultation. This retrospective reporting could lead to missed incidents. In general, the quality of evidence of adverse events following manipulative treatment is poor, and future studies should track possible adverse events throughout the study.

The information on sick leave was self-reported and retrospective, and this could result in a bias with respect to the reasons for, and duration of sick leave. Sick leave due to PGP and/or LBP was chosen to be our primary outcome measure because it represents a rather robust and easily measurable endpoint. Also, sick leave may indicate the level of pain experienced by these women, as well as the expense for the society. We intended to address sick leave caused by PGP and/or LBP only, but in many cases several different reasons for sick leave were reported. Nausea and fatigue are prominent disorders in the first trimester, and it seems that many women never return to work after having been on sick leave for some weeks. Because of this, we chose to exclude women on sick leave in week 1 − 18 when analyzing our primary outcome.

It is a strength to our study that we conducted a randomization process, enabling us to evaluate treatment results, as randomized studies have been particularly asked for in review articles when different PGP-treatments have been assessed [9, 11]. We believe that focusing on a specific subgroup of PGP is a strength to this study. Also, the RCT originates from a large prospective longitudinal study with follow-up during pregnancy and after delivery.

## Conclusions

In conclusion, we found no find statistically significant difference between the treatment and the control group in any of the outcome measures. The confidence intervals are wide, containing both positive and negative clinically relevant effects. Further studies on the effect of chiropractic management for specific subgroups of PGP are needed.

## Abbreviations

EQ-5 D: Eurocol-5D
LBP: low back pain
NRS: numeric rating scale
ODI: Oswestry Disability Index
PGP: pelvic girdle pain
PP: pelvic pain
RCT: randomized controlled trial
SD: standard deviation
